# A Complex Case of Highly Tortuous Abdominal Aorta Complicated with Infrarenal Aortoiliac Aneurysm

**DOI:** 10.2174/0115734056301340241105093932

**Published:** 2025-03-12

**Authors:** Dragan Piljic, Nail Sehic, Zijah Rifatbegovic, Haris Vukas, Fahrudin Sabanovic, Jus Ksela

**Affiliations:** 1 University Clinical Center Tuzla, Cardiovascular Surgery Clinic, Tuzla, Bosnia and Herzegovina; 2 Department of Vascular Surgery, University Hospital Zenica, Zenica, Bosnia and Herzegovina; 3 School of Medicine, University Clinical Center Ljubljana, Ljubljana, Slovenia

**Keywords:** Abdominal aortic aneurysm, Aortic tortuosity, Abdominal tortuous aorta, Aortobifemoral bypass, Endovascular aneurysm repair, Piljic method

## Abstract

**Background:**

Aneurysms, characterized by localized dilatation involving all three layers of the vascular wall, pose significant risks, with abdominal aortic aneurysm (AAA) being prevalent, particularly among the elderly. However, the cooccurrence of AAA with abdominal tortuous aorta (ATA) remains exceptionally rare.

**Case Report:**

We present the case of a 63-year-old male with an AAA extending into the iliac arteries, accompanied by ATA. Computed tomography revealed complex structural abnormalities, necessitating immediate surgical intervention. Due to the anatomical complexities, endovascular repair was not feasible, leading to a successful aortobifemoral bypass surgery using the Piljic method. The patient recovered well postoperatively, highlighting the efficacy of the chosen approach.

**Conclusion:**

While AAA is often treated with endovascular repair, ATA complicates this approach, underscoring the need for open surgery in such cases. Research on aortic tortuosity's role in rupture prediction and stress alleviation shows varied findings, necessitating additional studies. ATA may also hinder vascular catheter insertion, requiring alternative routes for interventions. Future research is imperative to develop tailored treatment strategies for patients with concurrent AAA and ATA, ensuring optimal outcomes.

## 1 INTRODUCTION

An aneurysm is a localized dilatation that involves all three layers of the vascular wall, typically resulting from the degeneration of the arterial media and elastic tissues. Aneurysms are classified morphologically as either fusiform, which involves the entire arterial circumference, or saccular, which affects only a portion. Fusiform aneurysms are the more common type [1]. An abdominal aortic aneurysm (AAA) is defined as a dilation of the abdominal aorta measuring 3.0 cm or greater, most commonly occurring in the infrarenal segment [2]. Key risk factors for AAA include male sex, smoking, age over 65, coronary artery disease, hypertension, previous myocardial infarction, peripheral arterial disease, and a family history of AAA [3]. In asymptomatic patients, abdominal ultrasonography is widely regarded as the gold standard for diagnosis and monitoring, while CT imaging is preferred for evaluating large, symptomatic, or ruptured AAAs [1]. Intervention is recommended for symptomatic aneurysms or in asymptomatic cases when the aneurysm diameter reaches 5.5 cm. Both endovascular aneurysm repair (EVAR) and open surgical repair are viable treatment options [4].

Arteries typically run straight, efficiently transporting blood to organs. However, they can become twisted or curved due to abnormal development or disease, presenting in various forms, such as curving, angulating, twisting, looping, and kinking, which are commonly identified in medical imaging [5]. Arterial tortuosity, defined by the abnormal twisting of one or more arteries, is associated with several factors, such as advanced age, female sex, hypertension, and other cardiovascular risks. Recent advances in medical genetics have also connected arterial tortuosity to a few rare genetic arteriopathies [6]. Arterial tortuosity increases the risk of aneurysm formation and dissection at any age, affecting both the aortic root and other parts of the arterial system [7]. While arterial tortuosity can be seen throughout the vascular system, the occurrence of a tortuous abdominal aorta (ATA) is exceedingly rare [8].

This study aims to underscore the complexity and clinical significance of ATA by presenting a rare case in which abdominal tortuosity progressed to a symptomatic abdominal aneurysm, necessitating urgent intervention. The case highlights the critical need for timely diagnosis of ATA, as well as the complexities of its management and the potential complications that may arise in clinical practice.

## 2 CASE REPORT

**Fig. (1):**
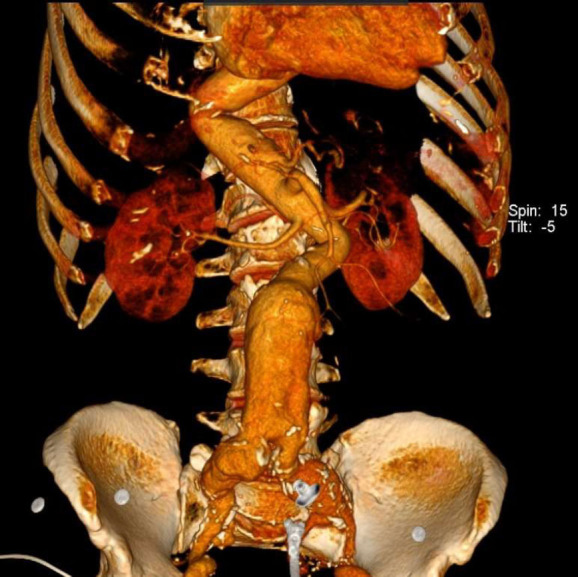
Unusual form of the suprarenal aorta with an aneurysm in the infrarenal aorta (8cm in transversal diameter) and an aneurysm in both common iliac arteries (right side – 5cm, left side – 4cm), and an aneurysm in the left internal iliac artery.

**Fig. (2A-D):**
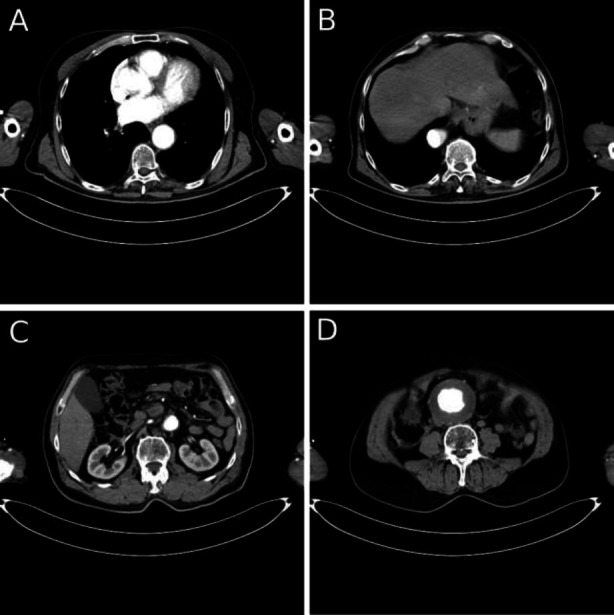
Unusual form of the suprarenal aorta with an aneurysm in the infrarenal aorta (axial).

A 63-year-old male patient was admitted to our clinic with an abdominal aortic aneurysm, diagnosed by transabdominal ultrasound. He presented with abdominal pain eight hours ago and a palpable abdominal mass. The patient reported experiencing paresthesia and bruising in both feet for the past four months. He had a medical history of irregularly controlled hypertension. Additionally, he underwent left hallux amputation (1 month prior), gastric surgery for a bleeding peptic ulcer under general anesthesia (7 years prior), and left inguinal hernia repair under spinal anesthesia (10 years prior). The patient was a regular smoker of 20 cigarettes a day until just one month before admission. He did not take his medication regularly. Computed tomographic angiography revealed multiple structural abnormalities of the aorta. Tortuosity originated in the descending thoracic aorta and continued into the abdominal segment without signs of rupture or dissection. The descending thoracic aorta, along with the suprarenal and infrarenal portions of the abdominal aorta, formed a Z-like (tortuous) configuration (proximal aortic angulation was 82°, and distal angulation was 85°). Aneurysmal dilatation began in the central part of the infrarenal abdominal aorta, with a maximum diameter of 8 cm and an aneurysmal neck measuring 3 cm. This dilatation extended to both common iliac arteries, measuring 4 cm on the left side and 5 cm on the right, and also involved the left internal iliac artery (Figs. **1** and **2**).

The complex, tortuous shape of the abdominal aorta, the aneurysm's angulated neck (measuring 2.2 cm), and the extension of the aneurysm into the iliac arteries—also exhibiting twisting shapes—precluded the possibility of endovascular aneurysm repair (EVAR). After consulting with the interventional radiologist, we concluded that open surgery was the only viable curative option. The patient underwent aortobifemoral bypass surgery utilizing the Piljic method [9]. The procedure was uneventful, and it involved meticulous dissection to access the abdominal aorta and both common femoral arteries. A 20/10mm bifurcated graft was utilized. The bypass graft was successfully anastomosed to both the abdominal aorta (end-to-end anastomosis) and the common femoral arteries (end-to-side anastomosis). During the surgery, we were able to preserve the right internal iliac artery, which is crucial for maintaining pelvic perfusion. Inferior mesenteric artery reimplantation was not performed due to the complex anatomy, insufficient retrograde flow, and the need to minimize operative time, focusing on ensuring optimal outcomes with the primary bypass.

**Fig. (3):**
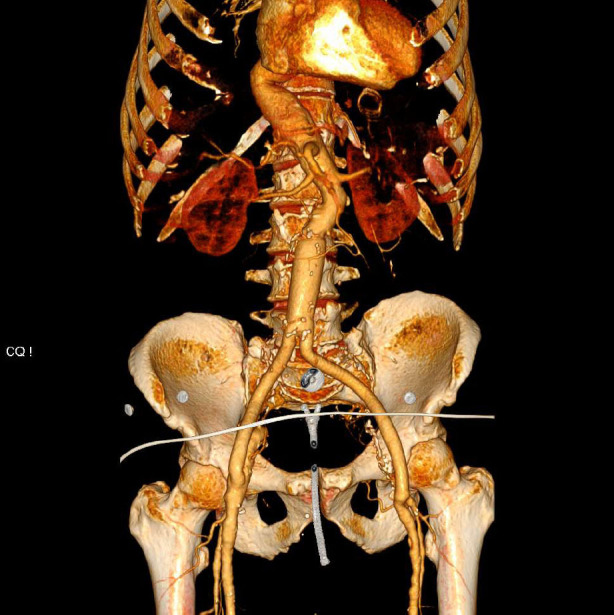
Postoperative computed tomography angiography with contrast showing complete sealing of the aneurysm and no endoleak detected.

In the postoperative period, the patient remained in good general health without any complications. A computed tomographic angiography was performed before discharge, showing complete sealing of the aneurysm, a well-positioned graft, and no evidence of endoleak or other complications (Fig. **3**). Consequently, the patient was discharged on the 4th postoperative day.

At the one-year follow-up, he continued to maintain relatively good health and showed no symptoms related to the aneurysm or surgical site. His regular monitoring revealed stable vital signs and no evidence of complications. Overall, the patient has been able to resume normal daily activities.

## 3 DISCUSSION

We presented a patient with a symptomatic AAA complicated by ATA. Due to the complex anatomical challenges, EVAR was not feasible, resulting in a successful aortobifemoral bypass surgery. This case highlights the importance of individualized treatment strategies in managing such complex scenarios.

Abdominal aortic aneurysm poses a significant global health burden, especially among the elderly, with a prevalence estimated at 3.67% in individuals aged 75 to 79 [10]. In contrast, abdominal tortuosity is exceptionally rare and has been documented in only a limited number of studies. As a result, there is a notable gap in the literature regarding the safety of abdominal surgery and the potential complications associated with this condition [11]. Given the rarity of the coexistence of both AAA and ATA, we found hardly any similar cases to guide our management strategy for this patient, further complicating the clinical decision-making process.

Aortic tortuosity is defined as the maximum lateral deviation from the centerline of the aorta, as observed on coronal CT scans [12]. The tortuosity of the abdominal aorta is an important factor in assessing rupture risk, alongside the maximal diameter of the aneurysm. Research on this topic presents varied findings: some studies suggest that tortuosity may slightly increase rupture risk, while others indicate it has no effect or may even provide protective benefits [13-15]. Hejazi *et al*. demonstrated in experimental studies that tortuosity can alleviate stress during inflation, potentially acting as a protective mechanism by offering alternative pathways for stress relief and thus preventing rupture [16]. Furthermore, a clinical study found that ruptured AAA cases exhibited less tortuosity than non-ruptured cases, suggesting that increased tortuosity could serve as a negative predictor for rupture [12].

ATA presents significant challenges, particularly regarding the insertion of vascular catheters through the abdominal aorta. While some studies, such as that by Chakravarthy *et al*., have reported successful catheter placement in cases of aortic abnormalities, these instances often occur under carefully controlled conditions and may not be applicable in all clinical scenarios [17]. Moreover, ATA has been linked to increased complications in patients undergoing transfemoral transcatheter aortic valve replacement, suggesting that alternative access routes may be necessary [18]. While EVAR has become a preferred option for treating AAAs, unfavorable aneurysm morphology—such as an angulated proximal neck—can hinder its application. Specifically, neck angulation exceeding 60° is a significant barrier. However, recent studies indicate that EVAR can be cautiously utilized even in anatomically challenging cases, as shown by Zeng *et al*. in a patient with a severely angulated neck [19-21]. In our case, the complex anatomy created by ATA and the aneurysm's morphology made such procedures highly risky, ultimately precluding the use of endovascular options like EVAR.

Open surgical repair often remains the only viable option for patients with AAA complicated by ATA, given the lack of consensus or established guidelines regarding the optimal approach and technique [22, 23]. In our case, we utilized the Piljic method, a novel surgical technique developed at our Medical Center specifically for abdominal aortic procedures. This method has demonstrated significant success in minimizing postoperative complications and reducing hospital stays [9, 24]. Our experience illustrates that the Piljic method is particularly effective in complex cases, as evidenced by our patient’s uneventful recovery and relatively swift discharge. This highlights the importance of adapting surgical techniques to the unique anatomical challenges posed by conditions like ATA, ensuring tailored interventions for improved patient outcomes.

## CONCLUSION

This case highlights the challenges of managing an AAA complicated by ATA. While EVAR is commonly employed for AAA treatment, the cooccurrence of both AAA and ATA often necessitates open surgical intervention. Our case emphasizes the significance of individualized, multidisciplinary approaches that adapt surgical techniques to the anatomical particularities of each case. It serves as a valuable example for managing rare and challenging surgical situations, which may particularly benefit surgeons facing similar scenarios. Furthermore, this case underscores the need for future studies to develop standardized treatment protocols for such complex clinical scenarios.

## Data Availability

All data generated or analyzed during this study are included in this published article.

## LIST OF ABBREVIATIONS

AAA: Abdominal Aortic Aneurysm
EVAR: Endovascular Aneurysm Repair
ATA: Abdominal Tortuous Aorta

## STANDARDS OF REPORTING

CARE guidelines were followed.
